# Prevalent Technique and Results of Hemorrhoidal Embolization

**DOI:** 10.3390/jcm11226631

**Published:** 2022-11-09

**Authors:** Silvia Buso Gil, María Dolores Ferrer Puchol, Jorge Solaz Solaz, Enrique Esteban Hernández

**Affiliations:** University Hospital of la Ribera, 46600 Alzira, Spain

**Keywords:** hemorrhoid, superior rectal artery, bleeding, coil embolization, particle embolization

## Abstract

Hemorrhoids are blood cushions located in the anus and lower rectum, acknowledged as a common cause of bleeding, which can reduce quality of life. The development of minimally invasive techniques such as endovascular embolization of superior rectal artery, “Emborrhoid technique”, is an effective treatment, with no pain or ischemic complications, and allows quick patient recovery. Our purpose is to describe the general technique and discuss the results of the current literature.

## 1. Introduction

Hemorrhoids (HD) are the most prevalent disease in the anorectal disorders field, representing 4% to 35% of the population; patients between 45 and 65 years old make up the highest incidence [1]. The classification of HD between internal and external comes from their location, whether above or below the dentate line (pectinate line). Internal HD often manifest with rectal bleeding, which reduces quality of life and may result in anemia [2,3].

They can be graded using the Goligher classification (GC) (Table 1), based on their degree of prolapse, and the French bleeding score (FBS) (Table 2), with a top score of 9, which implies the most intense bleeding [4].

Conservative management as dietary measures and topical medications can treat bleeding in the first instance [5,6,7,8]. However, 10% of all patients will need surgery such as conventional hemorrhoidectomy (CH) [9], circular anopexia [10], or stapled hemorrhoidopexy (SH) [11]. Over the years, less invasive techniques such as rubber band ligation (RBL) [12,13], sclerotherapy (SCL) [14,15,16], and infrared coagulation have been developed, allowing an outpatient setting and a quicker patient recovery, despite an increased recurrence. Recently, minimally invasive techniques based on the hyperflow of hemorrhoidal arteries, such as transanal hemorrhoidal dearterialization (THD) [17], doppler-guided hemorrhoidal artery ligation (DGHAL) [18,19], or its endovascular version, the “Emborrhoid technique” [20,21], have emerged, showing promising results and more patient comfort (Table 3) [22]. 

Among the main advantages of hemorrhoid embolization (HE) compared with other therapies is the identification and therefore, the occlusion of all branches dependent on the superior rectal artery (SRA) and any anastomoses with the middle rectal artery (MRA) and the inferior rectal artery (IRA), which reduces recurrence of bleeding. Compared to surgery, the endovascular approach avoids rectal manipulation, eliminating the risk of rectal trauma, allowing the preservation of anal continence. Coil and particle embolization of the SRA has been found to be a well-tolerated, effective, and safe technique [23,24,25,26,27,28].

Clear indications and patient selection have not been fully specified. The Italian society of colorectal surgery [22] indicates HE to patients suffering refractory symptoms from II and III HD degrees with contraindications to surgery (level of evidence 2, grade of recommendation C).

Some studies have analyzed the impact of HE on frail patients with severe cardiovascular, pulmonary, or neurological disease, which usually contraindicates the use of general anesthesia, finding excellent modifications in hemorrhoid bleeding, anemia, and patient’s quality of life after the endovascular procedure [23]. Patients with congenital or acquired bleeding disorders have shown good responses to HE, even without suspending antiplatelet or anticoagulant therapy [24,25]. Inflammatory bowel disease stays controversial as it appears as a particular indication or contraindication in the different literature [23,25,26]. Nowadays, it is also a suitable procedure for young surgical candidates who are averse to direct rectal manipulation [27].

As contraindications, we can find any situation that risks an endovascular procedure, like platelet count inferior to 50,000/μL, international normalized ratio (INR) superior to 1.5, allergy or intolerance to contrast media, non-available vascular access, or sepsis. Special situations that contraindicate HE are rectosigmoid resection, colorectal cancer, colonic angiodysplasia, or acute anorectal infection [23].

Considerable studies have been published on this subject, commonly using right transfemoral access (TFA). Nonetheless, many recent studies have demonstrated that transradial access (TRA) has faster ambulation and discharge but a higher radiation dose for not radial access trained specialists [26,28].

## 2. Anatomy

The rectum is mainly supplied by the SRA, a branch of the inferior mesenteric artery (IMA), and to a lesser extent by the MRA and IRA, branches of the internal iliac artery.

**Figure 1:**
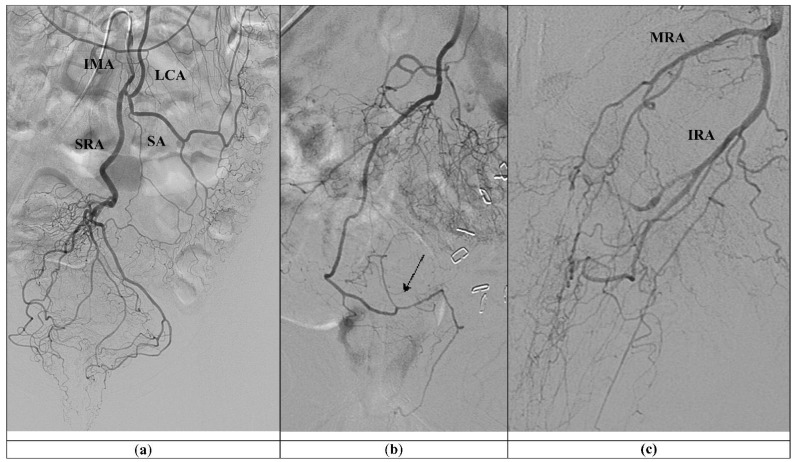
(**a**) Standard vascular anatomy of IMA: left colic artery (LCA), sigmoid arteries (SA), and type I superior rectal artery (SRA) are visualized after contrast injection. (**b**) Type II SRA: a right main trunk crosses and gives branches to the left (arrow). (**c**) Prominent left middle rectal artery (MRA) and inferior rectal artery (IRA) give the main blood supply to the left wall of the distal rectum.

The IMA origins from the anterior and left aspect of the abdominal aorta, immediately above the iliac bifurcation, at the level of the third lumbar vertebra, and bifurcates into the left colic artery, the sigmoid arteries, and the SRA, shown in Figure 1.

The anatomy of SRA was first described by Thomson in 1975, who found a common pattern in almost half of the cases, named as type I, where the main trunk divides into posterior-right and posterior-left branches and lateral-right and lateral-left branches, four branches in total. The rest of the cases were grouped into type II, where a right main trunk crosses and gives branches to the left, in type III, a left main trunk supplies the left side with a high contribution of the MRA to the corpus cavernosum recti (CCR), and type IV, the main trunk trifurcates, while in type V, the branches of the trifurcation do not reach the anal canal, because its main blood supply is done by the MRA [23,24].

## 3. Objective

This work’s primary goal is to describe the SRA embolization technique for treating internal hemorrhoids and review the indications, efficacy, outcomes, and complications published in the current literature.

## 4. General Technique

The “Emborrhoid technique” is generally indicated for patients with hemorrhoids type II or III and significant rectal bleeding, who have contraindications or refuse surgery, hence support by the coloproctology department must be facilitated.

Prior explanation of risk and benefit and informed consent must be obtained from each patient. Once fasting and adequate hemostasis are confirmed, the procedure is performed under aseptic conditions in a room equipped with a digital angiography. Conscious sedation is used, and local anesthetic is injected at the puncture site, which most commonly is the right femoral artery [25,26,27,28,29,30].

Simmons 2 5F (Radifocus; Terumo, Tokyo, Japan) is the most common catheter utilized to select the origin of the IMA [31,32]. In difficult catheterizations, it is also helpful to perform an inferior abdominal aortography with a Pig-tail 5F (Radifocus; Terumo, Belgium, Leuvem) (Merit; Utah, USA) and locate the c-arm in a lateral position [28].

Subsequently, there is the catheterization of the SRA with a 2.4 up to 2.7 F Progreat microcatheter^®^ (Radifocus; Terumo, Tokyo, Japan), Direxion microcatheter HI-FLO Bern shape or J shape^®^ (Boston Scientific; Marlborough, MA, USA) or RapidTransit microcatheter^®^ (Cordis Endovascular Systems, Miami Lakes, FL, USA). Angiography of the SRA branches and anastomoses with MRA and inferior rectal artery (IRA) can be acquired.

**Figure 2:**
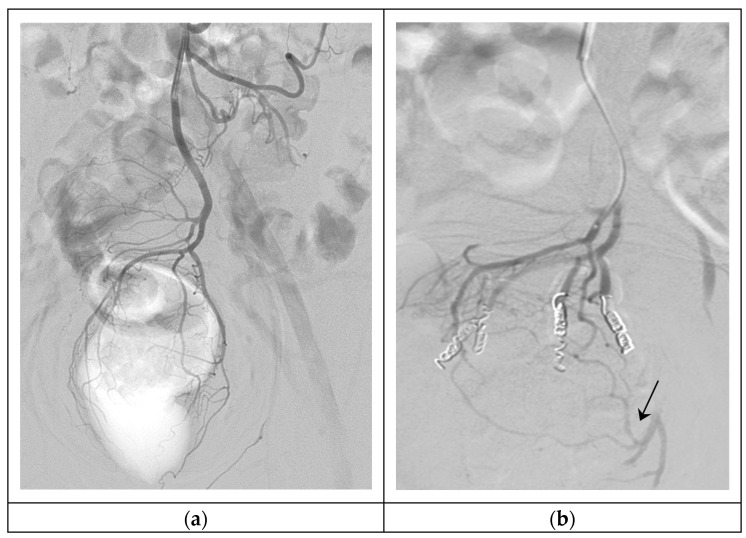
Hemorrhoidal embolization. (**a**) Arteriography of SRA, showing the vascular anatomy of the hemorrhoidal plexus (type I); (**b**) Super-selective embolization of four branches of the SRA with 300–500 μm particles and 3 mm 5 cm coils, persistent MRA anastomoses in the left part of the hemorrhoidal plexus (arrow).

Embolization of bilateral posterior and lateral branches can be performed with 2–3 mm pushable coils, such as Nester^®^ (Cook Medical; Bloomington, IN, USA), or detachable coils, such as Target^®^ (Stryker; Cork, Ireland) or Interlock (Boston Scientific; Marlborough, MA, USA). Some authors report bigger coil sizes of 4 to 7 mm with similar results [25,30]. Coils are the most often documented embolic material; regardless, the usage of particles and coils is preferred by some working groups. Previous injection of 300–500 μm polyvinyl alcohol (PVA) particles within the distal part of SRA branches, near the CCR; followed by coil embolization of the SRA branches themselves, may close hemorrhoid plexus more distally and obstruct persistent MRA anastomoses, as shown in Figure 2 (Table 4) [25,26,27,28,29,30,31,32]. A recent study showed that larger microspheres (900–1200 μm) have better long-term efficacy and no minor ischemic complications compared with smaller sizes [33]. Other embolization agents, like gel foam particles, have been proven effective [34].

It is also possible to use TRA with a 0.018-inch arterial micro-puncture set. It is advisable to puncture the left radial artery to minimize the risk of stroke. To avoid vasospasm, a “radial cocktail” (3000 IU of unfractionated heparin and 200 ugs of nitroglycerin or 2.5 mg of verapamil) is administered. Catheterization of SRA with 110 up to 150 cm-long multipurpose 4 or 5F catheter (Radifocus; Terumo, Tokyo, Japan) is advanced over a 0.035-inch × 260 cm-long glide wire, and then a 150 up to 175 cm-long microcatheter is used as TruSelect^®^ (Boston Scientific; Marlborough, MA, USA).

Patients with TFA are discharged home one day after the intervention, and patients with TRA after 6 h. A comparison of both accesses is made in Table 5 [28,30].

Patients are clinically followed up between 1 and 12 months. Technical success is considered when all the arterial network is properly embolized (Figure 2), and clinical success is when no rebleeding is observed between the first month and first year after embolization [25,26,27,28,29,30,31,32,33,34].

## 5. Outcomes and Safety in Current Literature

In 2006, Aigner performed transperineal Doppler ultrasound studies of the SRA in patients with HD and confirmed symptomatic hemorrhoids had increased flow velocities and bigger lumens [35].

Based on this foundation, in 2014, Vidal proposed the ‘‘Emborrhoid’’ technique, a new endovascular procedure consisting of selective embolization of the branches of SRA to treat hemorrhoidal bleeding. At one month, the technical and clinical success rates were 100% and 72%, respectively, and there were no major complications [20].

A study by Moussa et al. in 2017 was performed on 30 successive patients treated with this new technique, and 72% clinical success was obtained, in 17 patients after a single embolization and in 4 patients after second embolization. The level of protrusion did not change after arterial occlusion, but protrusion prevalence descended after embolization [4].

In 2016, Zakharchenco et al. reported a study of 40 patients treated with particles 300 μm in size and coils for embolizing distal arteries of SRA. The clinical success rate was 83% and 94% for patients with grade III and II HD. Particles may close hemorrhoid plexus more distally and obstruct MRA and even IRA anastomoses, found by authors in 20–40% of the procedures. Histopathology examination of the rectum and sphincterometry demonstrated normal mucosa and muscle contractility the first month after the treatment. They figured out that particles added to coil embolization did not induce ischemia and were a secure method [21].

M Ferrer et al. support distal branch embolization with PVA particles added to coil embolization by identifying MRA anastomoses in 70% of the cases and IRA in 20% of those. In patients with rebleeding, it was found that exclusively MRA restored the blood supply of the hemorrhoids, while the distal branches of the SRA were completely embolized. In their patients, proctoscopy one month after embolization showed a decrease in the hemorrhoid bulge with no proof of ischemia in the annus and rectum tissue [28].

In 2021, Makris et al. performed a meta-analysis of fourteen clinical studies, with a total of 362 cases. They found a substantial drop in the FBS after the treatment. In a deeper investigation, when coil-only embolization was compared with coil and 300–500 μm particles embolization, the average rebleeding rate was 21.5% versus 10.05%. No rectal complications were reported [36].

However, Moussa et al. suggested that clinical success and safeness were not significantly different when the 300−500 μm particles and the coils patient subgroup was compared with the coils-only patient subgroup [27].

A randomized study compared 500–700 μm, 700–900 μm, and 900–1200 μm microspheres sizes to perform SRA embolization. Smallest microspheres (500–700 μm) resulted in quicker bleeding and pain control, but a higher number of minor complications, such as small rectal and rectosigmoid ulcerations and small fibrotic scars. Largest microspheres (900–1200 μm) showed the best FBS improvement after twelve months, and no minor ischemic complications were observed. The 300–500 μm particles mentioned in most parts of the literature were not studied [33].

Recently, F.Tradi compared the safety of numerous embolic materials for the occlusion of SRA in healthy pigs. At autopsy, all the animals treated with Ethylene Vinyl Alcohol Copolymer (EVOH) developed necrosis of the distal part of the rectum. Embolization with coils had an acceptable tolerance but was revascularized by the internal iliac collateral arteries. Further, 500 μm microparticles were associated with satisfactory occlusion in the absence of rectal ischemia. These results may indicate that, for SRA occlusion, a super-selective embolization using particles might be more beneficial and as secure as coil treatment. EVOH should be a damaging embolization material for hemorrhoids. However, despite the fact that the vascular anatomy of pigs and humans are pretty comparable, these results may not be wholly reproducible [37].

Other embolization agents, like gel foam particles, have been proven to be effective [34].

This technique has an average of 30% recurrence of bleeding, which could be related to reperfusion by connections via the MRA or IRA, these arteries originate from the internal iliac arteries. Tradi et al. studied 25 patients who underwent SRA embolization. They reported that six cases showed prominent anastomosis with MRA [38].

Sun et al. detailed in their 23 patients study that 43% of them presented bilateral anastomoses between the SRA and IRA, while 13% had one-sided anastomoses [39].

In 2021, R Iezzi et al. published a pilot study where access by radial artery was used to perform embolization of bleeding hemorrhoids in 12 patients during a period of 4 months. The emborrhoid technique was successful in most of the patients. There were no crucial complications after the treatment, and all patients could be discharged after 6 h of the procedure [30].

M.Ferrer found that although TRA allows the patient to be discharged in a few hours, it also raises the procedure time and the radiation dose [28].

The review of M. Sirakaya in 2020 [31] and R. Talaie in 2022 [32] support the base of the embolization therapy in the different alternatives known for bleeding HD grade II and III. Indications of HE are still developing. The Italian society of colorectal surgery guidelines [22] supports HE in patients suffering low grade HD with contraindications to surgery or refractory symptoms.

Some studies have analyzed the impact of embolization on frail patients. In the report of Campennì [25], patients with severe cardiovascular or pulmonary disease or absolute contraindication to anesthesia were enrolled, finding excellent modifications in HD bleeding, anemia, and patient’s quality of life. The management of patients with congenital or acquired bleeding disorders is challenging. Patients with antiplatelet or anticoagulant therapy are included in most part of studies if they can stop medication temporarily; however, some authors, such as Venturini, suggest HE as a suitable treatment for patients with contraindications to suspension of antiplatelet or anticoagulation therapy [26]. Inflammatory bowel disease appears in the literature as a special indication or contraindication, depending on the working group, so further research is needed in this situation [25,27,28]. Recently, some authors offered HE to healthy patients that want to avoid surgery or rectal manipulation [40]. The aforementioned studies are exhibited in Table 6.

Technical success is considered when all arterial networks are properly embolized, with published rates between 100 and 90%. Technical failures in the series mentioned above were caused by IMA or SRA vasospasm impeding the catheterization of distal arteries, and one case of an infrarenal aortic aneurysm [26,38].

Clinical success is defined as an improvement in at least two points of the FBS, reduction of hematochezia, or discomfort during the first month after treatment. The published clinical success rates vary between 93% and 72%, with no significant complications. The patients who benefit most from the cessation of bleeding are the ones with anemia, presenting an increase in hemoglobin and hematocrit, which leads to a bigger increase in their quality of life. A reduction in discomforting symptoms and pain of HD is achieved with significant relief after embolization [20,21,23,24,25,26,27,28,33,34,38,39,40,41]. Most studies did not include validated quality of life (QoL) questionnaires [36]. Nonetheless, a recent systematic review and meta-analysis by Nguyenhuy [41] analyzed FBS, VAS, and as novel tool QoL scores, and found a post-procedural mean improvement of 2.66, 1.92, and 1.41, points respectively. Embolization has also been shown to reduce the size of hemorrhoids while maintaining sphincter function. Size reduction occurs within one month of treatment, with a quantifiable reduction in hemorrhoidal blood flow [31]. However, the degree of prolapse does not seem to change [31,36], with some publications finding a slight increase [42]. This is not unexpected given DGHAL and SCL add mucopexy when restoration of rectal mucosa is required [22].

Rebleeding is the main reason for clinical failure, mainly due to MRA anastomoses, which a second embolization can treat [26,27,28]. No major complications have been reported, but a single case of rectal sigmoid ischemia secondary to hemorrhoidal microparticle embolization in a 58-year-old patient [43]. Minor postprocedural complications as transient abdominal pain, tenesmus, and less commonly nausea and fever after the treatment were documented [31,32].

Hemorrhoidectomy is the gold-standard treatment for HD, as it reaches the highest clinical success among all the techniques; however, it has a considerable amount of complications and requires hospitalization [22]. Outpatient treatments are the techniques of choice in managing low-grade hemorrhoidal disease, with painless and quick recovery results. SCL is an effective and low pain procedure, and although there is a high recurrence rate, it is a low-cost and quick procedure that can be performed in a few minutes and repeated if needed. It has shown to be effective, even if patients with bleeding disorders do not discontinue anticoagulant therapy or undergo prior replacement therapy [13]. One interesting study by Gallo [44] makes a three-year follow-up of patients with HD grade II treated with 3% polidocanol foam, obtaining a final clinical success of 90.2% and an overall recurrence of 28%, with no problems in redo-sclerotherapy. No embolization studies have yet reported a follow-up of more than one year. It would be advisable to analyze the long-term outcomes of HE and compare them with other procedure techniques such as sclerotherapy. RBL appears to be more efficient in symptom resolution than SCL; nonetheless, SCL causes less postprocedural pain and minor complications. Some authors propose SCL as a first-line therapy and RBL for recurrent disease [14,15,16]. THD and DGHAL are minimally invasive techniques, with the same rationale as HE. The physiopathologic effect of DGHAL on hemorrhoidal hyperflow has been investigated by the study of Parello, with patients showing a significant decrease of main haemodynamic parameters compared with preoperative values [45]. There are no major differences in clinical success and recurrence rates when comparing HE and DGHAL or THD [22]. Future research is needed to compare efficacy and patient comfort between both treatments.

As limitations of the different published reports, we found that most of them are retrospective observational studies taken in one single-center with a small number of patients. Studies have heterogeneous patient selection and different follow-up timescales. Hence, further multicenter comparative investigations with longer follow-up times and samples with more patients are mandatory to determine the effectiveness, the indications, and the cost-effectiveness compared with the rest of the treatments.

## 6. Conclusions

Current literature suggests that hemorrhoid endovascular occlusion is a safe and painless alternative for patients with symptomatic hemorrhoidal disorder, finding coils in combination with larger particles to be the most effective and less damaging embolization agents.

## Figures and Tables

**Table 1:** Goligher classification.

Grade	Description
1	Bleeding with no protrusion
2	Protrusion spontaneously reduced
3	Protrusion needing digital reduction
4	Irreducible protrusion

**Table 2:** French Bleeding Score.

Frequency (0–4)	Never	0
	>1 per year	1
	>1 per month	2
	>1 per week	3
	>1 per day	4
Bleeding (0–3)	Never	0
	At wiping	1
	In the toilet	2
	On underwear	3
Anemia (0–2)	Never	0
	Without transfusion	1
	With transfusion	2

**Table 3:** Existing treatments for hemorrhoids.

Technique	Description	Indications	Advantages	Disadvantages
Conservative treatment: fiber, laxatives, phlebotonics	Improvement of stool consistency and vascular tone	Control of symptoms of HD grade I and II	Well tolerated with low adverse effects, symptoms relief	No changes in pathophysiology
Rubber band ligation (RBL)	With a proctoscope a rubber band is applied in each hemorrhoid to cause ischemia, followed by shrinkage and fibrosis	HD grade I, II, and III not responding to conservative management	Outpatient treatment, non-invasive procedure, more cost-effective, less recurrence rate than SCL and IRC	Relatively contraindicated in patients with anticoagulants, bleeding, or inflammatory disorders More painful than other outpatient procedures
Sclerotherapy (SCL)	The sclerosant injection into each hemorrhoid generates local inflammation and scarring	HD grade I, II, and III not responding to conservative management	Outpatient treatment, early improvement in bleeding and protrusion symptoms	Painful intraprocedural injection Mucosal ulceration up to 3.6% of patients Recurrence between 15–80% after the first year
Infrared coagulation	Infrared light applied directly to each hemorrhoid causes vessel coagulation followed by ischemia and scarring	HD grade I, II, and III not responding to conservative management	Outpatient treatment Coagulation of the internal hemorrhoid immediately visible Good patient improvement in I and II HD degree	Postprocedural pain and bleeding Insufficient data on long-term efficacy
Stapled hemorrhoidopexy (SH)	A trans-anal circular stapler sections circularly the hemorrhoidal network 4 cm above the dentate line.	HD grade III and IV (irreducible protrusion)	Non-excisional procedure Less operating time and hospital stay than CH	Higher recurrence than CH Early bleeding Early fecal urgency up to 8% of patients
Transanal hemorrhoidal dearterialization (THD) or Doppler-guided hemorrhoidal artery ligation (DGHAL)	A proctoscope and a Doppler transducer are used to recognize and ligate distal branches of the SRA above the dentate line. If combined with mucopexy, sutures are used to ensure the hemorrhoidal tissue in place.	II and III HD degree, and possibly IV in experienced surgeons	Low postprocedural pain and faster recovery than CH, SH, and RBL Added targeted mucopexy can be performed to treat prolapse	Higher recurrence rates than CH Tenesmus and pain if added mucopexy
Conventional hemorrhoidectomy (CH)	By an open (Milligan-Morgan) or closed (Ferguson) incision at the mucocutaneous junction the hemorrhoid cushion is exposed and excised	First choice for HD grade III and IV	Gold standard Lowest recurrence rate among all the techniques	Longer operating time and postoperative pain More loss of working days Fecal incontinence in 6% of the patients
Embolization of superior rectal artery (HE)	By angiography it is possible to identify and therefore occlude all the distal branches dependent on the SRA, lowering the vascular supply of internal hemorrhoids	HD grade II and III in patients with contraindications to surgery or refractory symptoms	Outpatient treatment Avoids rectal manipulation Preserves anal continence Quick and effective reduction of bleeding	Radiation No changes in prolapse Need for a second embolization in anatomy variants with high blood supply by MRA or IRA
Hemorrhoidal Laser Procedure	A Doppler transducer is used to detect the terminal branches of the SRA 2.5 cm above the dentate line, then a 980-nm diode causes shrinkage, thus reducing the blood supply	HD grade II and III IV if rectoanal repair or mucopexy is associated	Outpatient treatment It shares the foundation of DGHAL and THD but is less invasive and does not require general anesthesia	Postoperative bleeding and pain in up to 9% of cases
Laser hemorrhoidoplasty	After making a 1-mm opening, a fiber delivers 15 W pulses, inducing shrinkage of underlying tissues up to 5 mm in depth	HD grade II and III	It reduces postoperative pain and analgesics need if compared with CH	Recurrence up to 39% of patients

**Table 4:** Comparison between coil embolization vs. particles added to coil embolization.

Coils Alone	Particles and Coils
2–3 mm coils in SRA branches	Particles injection in the distal part of SRA branches, near the CCR, followed by coils
Proximal embolization	Proximal and distal embolization
Persistent MRA and IRA anastomoses	Obstruction of MRA and IRA anastomoses
Higher recurrence of bleeding, may need a second embolization	Lower recurrence of bleeding
Less postprocedural symptoms	Postprocedural rectal pain and tenesmus Asymptomatic and small superficial rectal ulcerations if particles < 900 μm

**Table 5:** Comparison between femoral and radial access.

TFA	TRA
Discharge after 24 h	Discharge on the same day
Most common and trained access, more material available	Need for training for most of interventional radiologists, longer procedures, and higher radiation dose (No difference for new specialists)
Contraindicated if antiplatelet or anticoagulant therapy or bleeding disorders	Possible if affected INR or level of platelets
Access site vascular complications	Lower access site vascular complications

**Table 6:** Main studies on SRA embolization.

Authors/ Study Year	Study Design	Patients Group	Technical/ Clinical Success (%)	Embolization Material	Complications
Vidal et al. [20] 2014	Prospective, single-center case series	14 patients with severe rectal bleeding due to HD grade II–IV, not suitable for surgical treatments	100/72	2–3-mm coils	1 episode of pain and tenesmus
Zakharchenko et al. [21] 2016	Prospective, single-center case series	40 patients with grade I–III HD	100/90	300 μm PVA particles, 3–5-mm coils	None
Moussa et al. [4] 2017	Retrospective, multicenter case series	30 patients with Chronic bleeding due to HD grade II, III, and IV	93/72	2–3-mm coils	1 episode of diarrhea
Tradi et al. 2018 [38]	Prospective, single-center case series	25 patients with chronic bleeding or pain due to HD grade II and III	96/64	2–3 mm coils	NR
Sun et al. 2018 [39]	Retrospective, single-center case series	23 patients with HD grade II and III	100/91.3	3 mm coils	Self-limited tenesmus in 34.78% of patients
Moussa et al. 2020 [27]	Retrospective case series	38 patients with chronic bleeding due to II and III HD degree	100/66	2–3 mm coils vs. 2–3 mm coils and 300–500 μm embospheres	Minor pain and bleeding in 35% of patients of particles group
Ferrer puchol et al. [28] 2020	Prospective case series	20 patients: 18 with chronic pain and bleeding due to HD grade II and III, with contraindications to surgery and 2 needing urgent embolization	90/83.4	300–500-μm PVA Particles and 2–3 mm coils	1 episode of IMA dissection 3 episodes of rectal heaviness and pain
Stecca et al. [29] 2021	Prospective, single-center case series	43 patients with symptomatic HD grade II and III	100/92	3–4-mm coils	1 episode of external hemorrhoid thrombosis 1 small hematoma in the puncture site
Iezzi et al. [30] 2021	Prospective case series	12 patients with symptomatic HD treated by radial approach	100/NR	4–7 mm coils	1 episode of ecchymosis and 1 mild postprocedural arm pain
Küçükay et al. [33] 2021	Prospective, single-center case series	42 patients with symptomatic I, II, III, IV HD degree	100/93	Tri-acryl-gelatin 500–700 μm, 700–900 μm, and 900–1200 particles	45% small superficial ulcerations 7% small rectosigmoid junction ulcerations 2% small fibrotic scar tissue
Wang et al. [34] 2021	Prospective, single-center case series	41 Patients with bleeding HD grade II and III and chronic anemia	100/87 vs. 88.9	2–3 mm coils + 350–560 μm gelfoam particles vs. 2–3 mm coils + 300–500 μm microparticles	NR
Moggia et al. 2021 [41]	Prospective, single-center case series	16 patients with HD (grade NR)	100/87.5	coils	None
Campennì et al. [25] 2022	Prospective, single-center case series	21 frail patients unsuitable for surgery with anemia due to HD	100/93	4–7 mm coils	3 patients needed transfusions during follow-up for recurrent hemorrhoidal bleeding
De Gregorio et al. [40] 2022	Prospective, single-center case series	21 patients with HD grade I, II, and III	100/80.9	2–5 mm coils	1 episode of radial hematoma 2 episodes of minor postprocedural tenesmus

## Abbreviations

HD, hemorrhoids; HE, hemorrhoidal embolization; GC, Goligher classification; FBS, French bleeding score; SRA, superior rectal artery; MRA, medial rectal artery; IRA, inferior rectal artery; IMA, inferior mesenteric artery; TFA, transfemoral access; TRA, transradial access; RBL, rubber band ligation; SCL, sclerotherapy; SHP, stapled hemorrhoidopexy; THD, transanal hemorrhoidal dearterialization; DGHAL, doppler-guided hemorrhoidal artery ligation, CH, conventional hemorrhoidectomy, PVA, polyvinyl alcohol; EVOH, Ethylene Vinyl Alcohol Copolymer; NR, non reported.
